# The Common Task

**Published:** 1907-04-13

**Authors:** 


### THE COMMON TASK.

### Hospitals and District Nurses.

There reach us almost simultaneously complaints of an opposite character, and each forcibly illustrating the want of elasticity in the present day chain of relief for the aid of the sick poor. From a hospital compelled frequently to discharge patients in a condition which makes their after-care by a trained nurse essential to their well-being, we learn that District Nursing Associations receive with scant courtesy requests for these cases to be attended to in their own homes, many replying not at all to the information sent. On the other hand, we learn that district nurses are frequently much hurt that patients are returned to their homes without any intimation being received which would enable the nurse to meet them on arrival and to continue the dressing and general treatment which the patients are probably too ignorant to carry out for themselves, and the neglect of which may seriously retard recovery. In such cases the District Nurse may not hear of the case till it has become aggravated for want of after-care. It is of the first importance to secure co-operation between district nurses and the hospitals, and we hope that without delay a general movement may be made to bring this about.

### The Battle for the Children.

It is the welfare of about 10 per cent. of the elementary scholars which is the stake in this battle, and the Rev. H. Iselin in this month's *Macmillan* tells us this is the most pressing problem of the day. How to turn these children into decent citizens without weakening parental control, how to substitute law for license, how to provide good food in place of wasteful messes, how to redeem the children from the disorders of their parents—this is the problem. Mr. Iselin sees hope in the action of the nurse, who is effecting what, he says, a century of "Mothers' Meetings" has failed to touch. But he strongly advocates a bracing attitude on the part of the State towards those parents whose neglect is bringing these difficulties upon the nation. It lies with district superintendents throughout the country to co-operate with the efforts being made to induce a sense of responsibility in parents, and to regard the work of the district nurse as far-reaching in its effects, designed for greater purposes even than the immediate relief of distress.

### The Nurse Training System in America.

The step recently taken in two prominent New York Hospitals of reducing the course of training for nurses from three years to two, is a curious comment on the effect of premature State Registration. In the New York Hospital the training school contains 85 nurses, divided into three grades. The Roosevelt Hospital has 70 pupil nurses, and seven fully-trained nurses, besides 16 male nurses. Both are general hospitals of high repute, and it is much to be regretted that they should have curtailed the term of training, since it can hardly fail to lower the standard of nursing and the status of the nurse, and this not only in the wards of these particular institutions. It is yet another proof that legislation, unless it is the outcome of a spontaneous and unanimous appeal from a united body, can effect very little in raising the standard of any profession. If imposed too soon, legislation has the effect of standardising a low minimum qualification. Should that error be avoided, the law often comes to be generally disregarded, while the profession, influenced by the irresistible laws of supply and demand, splits into two factions, one above and the other below the State requirements, both ignoring the statutory legislation, and on the whole doing very well without its proffered aid.

